# Phenotypic and morphometric characterization of local muscovy ducks raised in West Africa, Benin

**DOI:** 10.1371/journal.pone.0338829

**Published:** 2025-12-31

**Authors:** Daouda Libanio, Lionel Kinkpe, Tchilabalou Bouassi, Milognon Boris Behingan, Naqash Goswami, Ogechi E. Kadurumba, Anthony Pokoo-Aikins, Frederic Mankpondji Houndonougbo, Christophe Archille Armand Chrysostome

**Affiliations:** 1 Department of Animal Production, Faculty of Agricultural Science, University of Abomey-Calavi, Abomey-Calavi, Benin; 2 Department of Livestock Management, Breeding and Genetics, The University of Agriculture Peshawar-Pakistan, Peshawar, Pakistan; 3 Togolese Institute of Agricultural Research (ITRA), Lomé, Togo; 4 Department of Animal Breeding and Genetics, Sindh Agriculture University Tandojam, Pakistan; 5 Department of Animal Science and Technology, Federal University of Technology, Owerri, Nigeria; 6 US National Poultry Research Center, Toxicology and Mycotoxin Research Unit, USDA ARS, Athens, GA, 30605, USA; Universidade Federal de Mato Grosso do Sul, BRAZIL

## Abstract

The Muscovy duck (*Cairina moschata*) is a vital poultry species for rural livelihoods in Benin, yet no prior scientific study has documented its phenotypic and morphometric characteristics in the country. This study presents the first comprehensive characterization of Muscovy ducks raised across three agroecological zones of southern Benin, aiming to establish a foundational reference for sustainable breeding and conservation. A total of 406 adult ducks (260 females and 146 males) from 93 smallholder farms were assessed for both qualitative and quantitative traits. Significant sexual dimorphism was observed, with males exhibiting larger body dimensions than females (P < 0.05). A wide range of plumage patterns was recorded, dominated by white (58%), along with black, ash, and the first report of rare blue eye coloration. Beak and skin colours were mostly pink (66%) and white (73%), respectively; traits that may serve as important identifiers for local genetic lines. Notably, most morphometric traits varied significantly across colour morphs and agroecological zones, reflecting rich phenotypic diversity. Ducks with spotted white and grey plumage had superior measurements, while other colour groups excelled in specific traits such as thoracic cage width and leg length. Strong positive correlations were found between body weight and body length (r = 0.73), and leg length and chest height (r = 0.80). These results offer valuable insights for local producers and lay the groundwork for targeted selection, genetic improvement, and future molecular research on indigenous duck populations in Benin.

## 1. Introduction

Poultry farming plays an essential role in the agricultural economy of Benin, especially in rural agroecological zones, where it provides both food security and a valuable source of income [1]. Indigenous poultry species, such as Muscovy ducks (*Cairina moschata*), have become increasingly important due to their adaptability to local environmental conditions and economic value [2]. Muscovy ducks have a distinguished history in poultry farming. Initially domesticated in South America, it served as an important food source and a commodity of trade in pre-Columbian societies [3,4]. Following European colonization, it spread to other regions, including Asia, Africa, and Europe, where it became valued for its size, lean meat, and appealing flavor [5,6].

In Benin, Muscovy ducks are believed to have originated from South American stock [7]. These ducks are popular among consumers due to their lean, low-calorie and high-quality breast meat. Again, unlike chicken eggs, Muscovy duck eggs are mainly used for reproduction because the breed’s strong nesting ability enables them to lay 60–80 eggs per year under scavenging conditions [8]. Furthermore, they play a significant role in small-scale farming systems, where they are raised for cultural practices and economic benefits [3].

Phenotypic characterization of indigenous breeds is essential for enhancing local poultry production systems. Morphological traits, including body size, feather colour, and growth patterns, are crucial for developing breeding programs to improve productivity and meet consumer preferences. The first step involves using morphological or phenotypic characteristics (whether quantitative or qualitative) as indicators of animal genetic resources [9]. Similarly, the tolerance or vulnerability of poultry species to stressful environmental conditions could be associated with their phenotypic traits [10,11]. Although the Muscovy duck is a well-known duck breed and its economic importance is demonstrated among rural communities in developing countries, its phenotypic and genetic characterization in the tropics has been poor. This poor characterization delays the development of selection and breeding strategies.

Therefore, it is imperative to elucidate the phenotypic diversity inherent in Muscovy ducks adapted to specific local environments, as the information could fast-track genetic improvement programs.

To date, there is no study on the phenotypic characteristics of Muscovy ducks in Benin, leaving a significant gap in our understanding of the breed’s diversity and adaptability. In existing genetic diversity studies, the focus is primarily on exotic poultry breeds’ production, growth performance, and socio-economic factors associated with their breeding [12,13]. Research findings on Muscovy ducks in similar agroecological zones have demonstrated the importance of morphological traits when assessing the adapt-ability and performance of the breed under local conditions [14,15]. Given Muscovy ducks’ crucial role in food security and poverty alleviation, addressing this data gap is critical for promoting sustainable duck farming practices and conserving indigenous genetic resources in the region.

The study aimed to determine the phenotypic characterization of Muscovy ducks in Benin, focusing on the three distinct agroecological zones in the country that are most developed for duck breeding. By evaluating both qualitative and quantitative traits, the study will explore the influence of environmental factors and sexual dimorphism on phenotypic variability, providing insights into the breed’s adaptation and potential for enhanced breeding strategies.

## 2. Materials and methods

### 2.1. Ethics

The Faculty of Agronomic Science (**FSA**) and the University of Abomey-Calavi ethics and scientific committees approved this investigation, which was conducted in the Zootechnical laboratory and experimental unit.

### 2.2. Study areas

**Fig 1:**
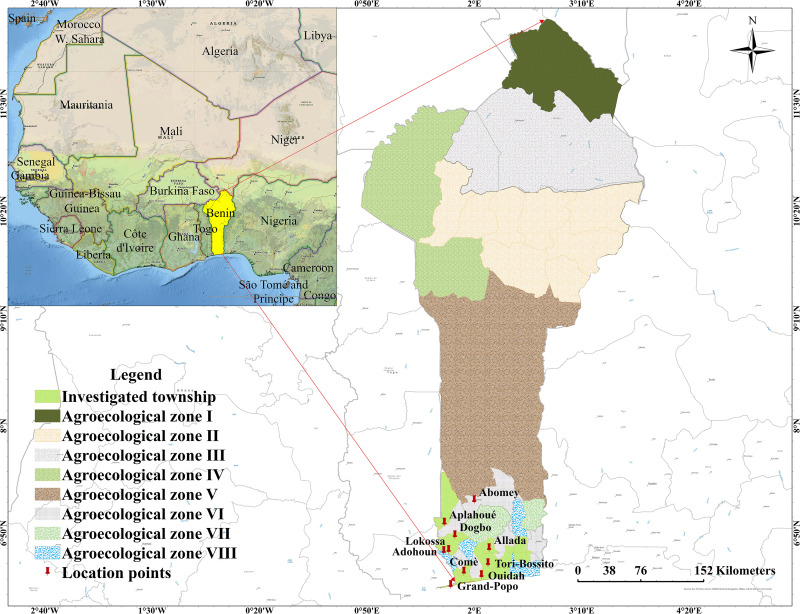
Localization of study areas].

The study was conducted in southern Benin across three distinct Agro-ecological zones. Fig 1 illustrates the location of these zones along with their respective temperature profiles. The selection criteria and rationale for these Agro-ecological zones are comprehensively outlined in our previous study [8].

Briefly, through a non-probability sampling approach, a survey was conducted across three agroecological zones (where duck breeding activities were most dominant) in southern Benin. All data collection was conducted with prior consent from participants.

### 2.3. Data collection and sample size

A total of 406 mature Muscovy ducks, comprising 260 females and 146 males, represented the study population. To reduce human error and ensure consistency, all qualitative and quantitative measurements were conducted by the same trained individual, who adhered to the established protocols outlined by the Food and Agriculture Organization [9]. These protocols provided comprehensive guidelines for sampling strategies, standardized data collection methods, and recommended statistical approaches. Phenotypic characterization was performed integrating both qualitative and quantitative traits as presented in Table 1 below.

**Table 1:** Morphological and Qualitative Trait Descriptions for Indigenous Ducks.

Trait	Description	Measurement Method	Unit of Measurement
Qualitative Traits	Eye Colour, Beak Colour, Plumage Colour, Plumage Morphology, Shank Colour	Visual inspection	–
Head Length	Distance between the lacrimal bone and the most prominent point of the occipital bone.	Measurement with a graduated flexible tape	cm
Neck Length	Distance between the cephalic margins of the coracoids and the occipital condyle.	Measurement with a graduated flexible tape	cm
Body Length	Distance between the atlas and the posterior end of the ischium.	Measurement with a graduated flexible tape	cm
Wing Length	Distance between the terminal ends of the phalanx (digit III) and the starting point of the humerus.	Measurement with a graduated flexible tape	cm
Shank Length	Distance between the hock joint and the foot pad.	Measurement with a graduated flexible tape	cm
Toe Length	Distance between the tip of the claw or toenail and the longest toe.	Measurement with a graduated flexible tape	cm
Thigh Length	Distance between the pelvic joint and the hock joint.	Measurement with a graduated flexible tape	cm
Bill Length	Length of the bill from the bill tip to the bill base.	Measurement with a graduated flexible tape	cm
Breast Length	Length of the area of the breastbone.	Measurement with a graduated flexible tape	cm
Breast Width	Distance between both lateral caudal processes on opposite ends of the body.	Measurement with a graduated flexible tape	cm
Body Weight	Live weight of the duck.	Weighing scale	kg

### 2.4. Statistical analysis of data

The data collected were systematically organized, cleaned, and processed to ensure accuracy and usability before analysis, which was conducted using R software (R Core Team, 2021) and Python. Descriptive statistics, including sums, frequencies, and means, were employed to summarize the data, with variables such as sex and Agro-ecological zones treated as independent factors.

The analysis model used was:


$${\text{Y}}_{\text{ijk}}\;=\;\mu +\;A_{i}+\;B_{j}+\;{\left(AB\right)}_{ij}+\;e_{ijk}$$

Where:

- *Y*_*ijk*_: Represents the quantitative trait of the Muscovy duck in the *i*^*th*^ agroecological zone (*i* = 3, ZAE8, ZAE6, ZAE5) and the *j*^*th*^ sex (*j* = 2, male and female).
- μ: Is the overall population mean for the respective quantitative trait.
- *A*_*i*_: Is the effect of the *i*^*th*^ agroecological zone.
- *B*_*j*_: is the effect of sex *j*^*th*^ (j = 2, male and female).
- *(AB)_ij_*: is the interaction effect between agroecology and sex.
- *e*_*ijk*_ is the residual error [16].

To explore differences in phenotypic traits across various agroecological contexts and sexes, the study utilized Analysis of Variance (ANOVA) to assess significant differences in the means of quantitative traits across different groups. To identify where these differences occurred, a post-hoc Tukey’s Honest Significant Difference (HSD) test was applied for multiple comparisons. This method allowed for the determination of which specific groups differed significantly in terms of their phenotypic traits. To determine the relationship between body weight (BW) and other linear body measurements, Pearson correlation analysis was deployed. Furthermore, Principal Component Analysis (PCA) tried to reduce the dataset’s dimensionality while retaining as much variance as possible, facilitating an understanding of the underlying patterns.

## 3. Results

### 3.1. Influence of sex and regional variability in morphometric characterization of ducks across agroecological zone (ZAE)

The morphometric diversity of ducks across various ZAE in Benin, categorized by sex, reveals significant variations in phenotype and sex (Tables 2 and 3), with males consistently exhibiting higher values across all measured parameters (P < 0.05).

**Table 2:** Diversity of physical characteristics of ducks in the different ZAE by sex.

	Agroecological Zone							
	Zone 5		Zone 6		Zone 8		Overall	
	Female	Male	Female	Male	Female	Male	Female	Male
**Body Weight**	1.80 ± 0.2^a^	2.7 ± 0.28^b^	1.87 ± 0.51^a^	3.16 ± 0.82^b^	1.75 ± 0.22^a^	2.67 ± 0.49^a^	1.80 ± 0.02^a^	2.80 ± 0.05^b^
**Body Length**	48.83 ± 3.43^ab^	51.75 ± 2.87^b^	45.01 ± 8.57^a^	52.39 ± 8.70^b^	43.82 ± 4.50^a^	50.16 ± 6.52^a^	45.88 ± 0.42	51.43 ± 0.64^b^
**Trunk Length**	29 ± 2^ab^	35.25 ± 2.63^bc^	29.42 ± 4.86^a^	33.18 ± 3.42^c^	30.19 ± 3.50^a^	33.51 ± 3.58^a^	29.79 ± 0.26^a^	33.39 ± 0.29^b^
**Wattle Length**	10.27 ± 1.50^abc^	13.25 ± 2.06^c^	6.25 ± 2.29^a^	6.89 ± 3.50^c^	8.24 ± 4.32^b^	10.16 ± 4.20^a^	7.34 ± 0.24^a^	8.54 ± 0.34^b^
**Beak Length**	3.3 ± 0.25^ab^	3.82 ± 0.23 cd	4.27 ± 1.25^ac^	4.53 ± 0.77^bd^	4.01 ± 0.66^a^	3.74 ± 0.83^a^	4.12 ± 0.06^a^	4.15 ± 0.07^a^
**Head Length**	6.43 ± 1.33^abc^	8.35 ± 0.43^bc^	5.79 ± 1.72^a^	7.36 ± 1.73^c^	6.77 ± 1.67^ab^	8.09 ± 1.22^a^	6.29 ± 0.10^a^	7.71 ± 0.12^b^
**Neck Length**	7.83 ± 1.17^a^	10.12 ± 1.94^b^	13.07 ± 3.87^a^	15.66 ± 4.17^b^	9.62 ± 1.72^a^	11.46 ± 2.36^a^	11.23 ± 0.21^a^	13.61 ± 0.33^b^
**Head Width**	4.35 ± 0.74^abc^	4.57 ± 0.41^c^	4.53 ± 1.17^ab^	4.99 ± 1.03^bc^	3.71 ± 0.94^a^	4.33 ± 0.64^a^	4.12 ± 0.06^a^	4.68 ± 0.07^b^
**Thoracic Cage Width**	9.91 ± 1.96^ab^	10.25 ± 3.06^c^	13.37 ± 2.49^a^	15.57 ± 3.06^bc^	10.25 ± 2.73^ab^	11.32 ± 3.56^a^	11.92 ± 0.19^a^	13.50 ± 0.32^b^
**White Feathers Length**	21.5 ± 1.33^a^	21.75 ± 2.98^b^	21.80 ± 3.86^a^	25.27 ± 2.77^ab^	20.2 ± 3.44^a^	20.59 ± 3.43^a^	20.99 ± 0.22^a^	23.06 ± 0.31^b^
**Shank Length**	11.41 ± 2.28^abc^	12.25 ± 2.21^bc^	9.94 ± 3.84^ab^	8.82 ± 3.15^c^	10.45 ± 1.77^a^	11.58 ± 1.94^a^	10.23 ± 0.18^a^	10.16 ± 0.24^a^
**Foot Diameter**	6.75 ± 0.61^ab^	9.32 ± 0.39^bc^	7.01 ± 2.98^ab^	7.31 ± 3.79^c^	6.11 ± 2.72^a^	8.21 ± 2.99^a^	6.44 ± 0.17^a^	7.77 ± 0.28^b^
**Tarsus Length**	6.17 ± 0.40^a^	7.5 ± 0.57^ab^	7.45 ± 2.76^ab^	7.01 ± 3.43^b^	6.68 ± 1.04^a^	7.51 ± 1.34^a^	7.03 ± 0.12^a^	7.25 ± 0.21^b^
**Leg Length**	5.67 ± 0.51^a^	6.75 ± 0.5^bc^	13.15 ± 4.09^ab^	13.82 ± 4.70^c^	5.99 ± 0.96^a^	6.75 ± 0.85^a^	9.40 ± 0.28^a^	10.43 ± 0.40^b^
**Keel Length**	19.66 ± 4.80^abc^	18 ± 7.5^bc^	23.30 ± 6.83^ab^	23.89 ± 8.00^c^	16.17 ± 5.08^a^	19.13 ± 7.33^a^	19.65 ± 0.42^a^	21.58 ± 0.66^b^
**Chest Height**	5.33 ± 0.51^ab^	7 ± 0.81^d^	11.59 ± 2.12^a^	13.96 ± 3.13 cd	7.10 ± 1.95^bc^	7.41 ± 1.73^a^	9.20 ± 0.18^a^	10.81 ± 0.34^b^
**Forearm Length**	12 ± 2.97^ab^	13.25 ± 2.59^b^	12.47 ± 2.36^ab^	13.49 ± 2.41^ab^	11.06 ± 1.24^a^	11 ± 3.29^a^	11.75 ± 0.15^a^	12.35 ± 0.25^b^
**Wing Length**	26.16 ± 2.63^ab^	28.25 ± 3.02^b^	28.89 ± 27.39^a^	33.87 ± 10.57^ab^	27.37 ± 8.68^a^	27.27 ± 9.80^a^	28.07 ± 1.23^a^	30.73 ± 0.88^b^
**Tarsus Diameter**	1.25 ± 0.122^a^	1.26 ± 0.09^bc^	3.64 ± 4.33^ab^	2.58 ± 1.72^c^	1.35 ± 0.81^a^	1.60 ± 0.32^a^	2.44 ± 0.20^a^	2.11 ± 0.11^b^
**Webbing Width**	4.25 ± 0.27^abc^	5 ± 0.57^bc^	4.21 ± 0.88^ab^	4.54 ± 0.73^c^	4.16 ± 0.58^a^	4.91 ± 0.70^a^	4.19 ± 0.04^a^	4.72 ± 0.06^b^
**Thoracic Circumference**	18.67 ± 1.63^a^	22 ± 1.41^c^	27.40 ± 7.11^ab^	31.16 ± 7.53^bc^	22.40 ± 5.51^a^	24.89 ± 5.00^a^	24.70 ± 0.42^a^	28.07 ± 0.59^b^
**Shank Circumference**	4.41 ± 0.49^ab^	5.12 ± 0.25^c^	5.90 ± 3.19^ab^	6.63 ± 0.94^bc^	4.97 ± 1.60^a^	5.47 ± 1.19^a^	5.40 ± 0.15^a^	6.07 ± 0.09^b^
**Beak Width**	1.91 ± 0.15^a^	2.2 ± 0.11^c^	2.39 ± 0.49^ab^	2.65 ± 0.39^bc^	2.44 ± 2.33^a^	2.31 ± 0.22^a^	2.40 ± 0.10^a^	2.48 ± 0.03^b^

This table summarizes the diversity of morphological traits in ducks, stratified by sex, across three principal Agro-ecological zones in southern Benin: The Central Cotton Zone (ZAE5), the “Terre de Barre” Zone (ZAE6), and the Fisheries Zone (ZAE8).

**Table 3:** Least squares mean and standard error of live body weight (kg) and body measurements of Muscovy ducks according to phenotype and sex.

	Phenotype										P-Value	Sex		P-value
	white green black	White	Spotted white and black	ash	Black	Gray and black mottled	Spotted white and gray	black piebald	Green, black, and white mottled	white green blue		Female	Male	
**BW**	2.44 ± 0.32	2.29 ± 0.18	2.13 ± 0.19	2.07 ± 0.18	1.90 ± 0.25	1.87 ± 0.47	1.85 ± 0.56	1.76 ± 0.32	1.70 ± 0.77	1.60 ± 0.77	0.1482	1.81 ± 0.39^a^	2.92 ± 0.71^b^	2.2e-16 ***
**Body Leng**	42.00 ± 11.40 ^abc^	48.54 ± 6.70 ^ab^	45.72 ± 8.74 ^abc^	41.42 ± 8.40 ^ac^	41.11 ± 7.43 ^ac^	44.67 ± 6.81 ^abc^	59.00 ± 1.41 ^a^	46.50 ± 6.68 ^abc^	46.00 ± 0.12 ^abc^	27.00 ± 0.15 ^bc^	1.83e-07 ***	44.51 ± 6.78^a^	51.37 ± 7.72^b^	2.2e-16 ***
**Trunk_Length**	30.00 ± 5.53 ^a^	31.33 ± 4.05^a^	31.04 ± 4.50 ^a^	30.00 ± 5.29 ^a^	29.94 ± 4.61 ^a^	31.00 ± 6.56 a	35.50 ± 3.54 ^a^	30.75 ± 3.77 ^a^	29.00 ± 0.15 a	22.00 ± 0.86^a^	0.30	29.79 ± 4.19^a^	33.39 ± 3.47 ^b^	2.2e-16 ***
**Wattle_Length**	3.24 ± 3.88 ^bc^	9.33 ± 2.65 ^a^	5.87 ± 4.16 ^abc^	2.25 ± 3.77 ^c^	4.49 ± 5.38 ^bc^	7.33 ± 6.35 ^abc^	13.00 ± 0.00 ^a^	8.70 ± 3.99 ^ab^	6.50 ± 0.12 ^abc^	---------	2.2e-16 ***	7.34 ± 3.84^a^	8.55 ± 4.20^b^	0.003476 **
**Beak_Length**	5.06 ± 0.73 ^ab^	3.93 ± 0.73 ^c^	4.41 ± 1.16 ^b^	4.86 ± 0.79 ^b^	4.47 ± 1.05 ^b^	4.00 ± 0.50 ^bc^	2.35 ± 1.77 ^c^	3.74 ± 0.35 ^c^	6.80 ± 0.11^a^	4.40 ± 0.05 ^bc^	1.47e-09 ***	4.12 ± 1.00	4.16 ± 0.88	0.7236
**Head_Length**	8.24 ± 2.12 ^a^	6.33 ± 1.43 ^b^	7.28 ± 1.99 ^a^	8.27 ± 1.97 ^a^	8.47 ± 1.98 ^a^	8.70 ± 2.07 ^a^	7.25 ± 0.35 ^ab^	6.63 ± 1.55 ^ab^	4.50 ± 0.08 ^b^	6.20 ± 0.02 ^b^	9.32e-11 ***	6.30 ± 1.75^a^	7.72 ± 1.54^b^	3.52e-15 ***
**Neck_Length**	12.59 ± 3.16^ab^	11.70 ± 3.94 ^b^	13.26 ± 3.71 ^a^	12.28 ± 3.59 ^ab^	12.21 ± 3.07 ^ab^	11.00 ± 2.65 ^b^	12.00 ± 1.41 ^ab^	8.70 ± 1.70 ^b^	13.00 ± 0.09 ^ab^	10.00 ± 0.06 ^b^	0.02 *	11.23 ± 3.44^a^	13.61 ± 4.03^b^	8.18e-10 ***
**Head_Width**	4.01 ± 1.43 ^b^	4.52 ± 0.87 ^a^	4.09 ± 1.04 ^b^	3.82 ± 2.09 ^b^	3.89 ± 1.84 ^b^	3.53 ± 0.50 ^b^	4.50 ± 0.71 ^ab^	4.05 ± 1.07 ^b^	4.50 ± 0.05 ^ab^	3.30 ± 0.32 ^b^	0.01 **	4.12 ± 1.13^a^	4.69 ± 0.92^b^	3.30e-07 ***
**Th_Cage_Width**	14.93 ± 2.67 ^a^	11.86 ± 3.63 ^b^	13.61 ± 3.18 ^a^	14.52 ± 4.44 ^a^	12.67 ± 5.58 ^ab^	10.00 ± 4.36 ^b^	11.00 ± 2.83 ^b^	11.75 ± 3.06 ^b^	12.00 ± 0.86 ^ab^	13.00 ± 0.41 ^ab^	0.00 ***	11.93 ± 3.14^a^	13.50 ± 3.94^b^	1.31e-05 ***
**W_Feathers_Lgth**	24.13 ± 1.89 ^a^	20.89 ± 3.83 ^b^	23.10 ± 3.58 ^ab^	23.68 ± 3.61 ^ab^	23.06 ± 3.61 ^ab^	20.00 ± 2.00 ^b^	22.50 ± 4.95 ^ab^	20.88 ± 5.22 ^b^	24.00 ± 0.02 ^ab^	23.00 ± 0.05 ^ab^	3.29e-05 ***	21.00 ± 3.70^a^	23.06 ± 3.86^b^	1.72e-07 ***
**Shank_Length**	10.56 ± 3.19 ^ab^	9.99 ± 2.66 ^b^	9.87 ± 3.15 ^b^	12.42 ± 3.64 ^a^	12.00 ± 4.36 ^a^	12.00 ± 1.73 ^ab^	11.25 ± 1.06 ^ab^	10.94 ± 1.15 ^ab^	8.50 ± 1.25 ^b^	9.60 ± 0.15 ^b^	0.01 **	10.24 ± 2.96	10.17 ± 2.98	0.8218
**Foot_Diameter**	3.15 ± 3.01^b^	8.12 ± 1.84a	5.91 ± 3.75^a^	3.23 ± 4.01^b^	3.63 ± 3.58^ab^	5.73 ± 4.05^ab^	8.00 ± 1.41^a^	7.13 ± 2.64^a^	9.00 ± 0.01^a^	1.20 ± 0.03^b^	2.2e-16 ***	6.56 ± 2.85^a^	7.78 ± 3.43^b^	0.0001 ***
**Tarsus_Length**	4.90 ± 2.33^c^	7.51 ± 2.36^a^	6.69 ± 2.06^bc^	6.26 ± 2.31^bc^	6.02 ± 1.84^bc^	6.67 ± 1.15^bc^	7.00 ± 2.12^abc^	7.38 ± 1.53^ab^	6.40 ± 0.12^bc^	5.90 ± 0.10^bc^	0.01 **	7.04 ± 2.08	7.26 ± 2.64	0.361
**Leg_Length**	9.78 ± 5.88^abc^	9.93 ± 4.85^ab^	10.65 ± 4.72^a^	7.96 ± 3.89^abc^	6.26 ± 1.73^ac^	6.33 ± 0.58^abc^	6.00 ± 0.00^ac^	8.60 ± 4.35^abc^	15.00 ± 0.02^a^	5.20 ± 0.01^ac^	0.01 **	9.40 ± 4.62^a^	10.44 ± 4.93^b^	0.03518 *
**Keel_Length**	16.85 ± 5.49^b^	20.94 ± 7.11^ab^	20.67 ± 7.55^ab^	15.21 ± 7.47^b^	17.22 ± 6.83^b^	16.67 ± 6.43^b^	28.50 ± 12.02^a^	20.06 ± 10.32^ab^	25.00 ± 0.01^ab^	15.00 ± 0.002	0.02 *	19.65 ± 6.93^a^	21.58 ± 8.02^b^	0.0113 *
**Chest_Height**	12.78 ± 3.48^a^	9.33 ± 3.72^b^	10.97 ± 3.21^ab^	10.35 ± 2.48^ab^	8.56 ± 2.62^b^	7.00 ± 1.73^b^	5.75 ± 1.77^b^	9.11 ± 3.52^b^	11.00 ± 0.01 ^ab^	10.00 ± 0.02^ab^	0.00 ***	9.21 ± 3.06^a^	10.81 ± 4.16^b^	1.12e-05 ***
**Forearm_Length**	13.16 ± 2.55^a^	11.61 ± 2.89^ab^	12.80 ± 2.49^ab^	12.40 ± 2.90^ab^	11.47 ± 1.37^ab^	13.00 ± 2.00^ab^	13.50 ± 0.77^a^	10.26 ± 2.36^ab^	15.00 ± 0.03^a^	13.00 ± 0.04^ab^	0.01 *	11.76 ± 2.55^a^	12.36 ± 3.09^b^	0.03505 *
**Wing_Length**	40.38 ± 9.27^ab^	25.70 ± 18.93^ac^	32.52 ± 12.18^abc^	40.68 ± 10.46^a^	39.78 ± 11.98^ab^	36.00 ± 13.89^abc^	30.00 ± 1.41^abc^	20.14 ± 11.98^ac^	30.00 ± 0.18^abc^	42.00 ± 0.01^a^	2.56e-05 ***	28.07 ± 19.87	30.74 ± 10.68	0.1334
**Tarsus_Diameter**	2.67 ± 2.36	2.38 ± 1.96	2.13 ± 1.64	3.66 ± 9.61	1.47 ± 0.94	1.27 ± 0.58	1.35 ± 0.35	1.72 ± 0.87	6.00 ± 0.01	0.8 ± 0.08	0.33	2.44 ± 3.25	2.12 ± 1.35	0.2496
**Webbing_Width**	4.12 ± 1.00^ab^	4.61 ± 0.67^a^	4.07 ± 0.74^ab^	3.75 ± 1.00^ab^	3.95 ± 0.87^ab^	4.03 ± 0.80^ab^	4.75 ± 0.35^a^	4.3 ± 0.54^ab^	4 ± 0.03^ab^	2.8 ± 0.01^b^	2.31e-10 ***	4.19 ± 0.74^a^	4.72 ± 0.74^b^	1.22e-11 ***
**Thora_Circ**	36.62 ± 6.07^a^	24.23 ± 6.28 ^ab^	28.22 ± 7.31^ab^	33.84 ± 4.8^a^	28.61 ± 9.08^ab^	23.67 ± 6.43^ab^	18 ± 2.82 ^b^	20.62 ± 4.75^b^	31 ± 0.02^ab^	36 ± 0.04^a^	3.07e-11 ***	24.71 ± 6.80^a^	28.08 ± 7.16^b^	3.53e-06 ***
**Shank_Cir**	7.30 ± 1.09^ab^	5.16 ± 1.08^c^	6.31 ± 3.55^b^	7.34 ± 2.28^a^	6.33 ± 1.87^ab^	5.33 ± 1.53^bc^	4.75 ± 1.06^c^	5.36 ± 1.58^bc^	5.00 ± 0.10 ^c^	6.20 ± 0.50 ^bc^	4.51e-06 ***	5.40 ± 2.53^a^	6.07 ± 1.21^b^	0.002751 **
**Beak_Width**	2.39 ± 1.23	2.39 ± 0.12	2.62 ± 0.35	2.42 ± 0.21	2.23 ± 0.02	2.37 ± 0.02	2.15 ± 1.95	2.25 ± 0.32	2.30 ± 0.37	2.20 ± 0.21	0.97	2.40 ± 1.69	2.49 ± 0.37	0.5577

As revealed in Table 2, Zone 6 recorded the highest morphometric values for most traits measured in males with the exception of the tarsus diameter which showed a higher mean in females (3.64 ± 4.33). Other traits followed a consistent pattern, with males displaying larger measurements across board. Distinct regional differences were also evident in Zone 5, where significant high values were observed for trunk length, wattle length, head length, shank length, foot diameter, and webbing width (P < 0.05). Similar to Zone 6, males predominated in most of these characteristics, though females exhibited a higher mean in shank length. Conversely, Zone 8 showed the lowest values for nearly all parameters except thoracic circumference, which was recorded as the highest for this zone. Interestingly, the thoracic circumference was smaller in females than in males in this zone.

The overall average body weight (BW) for the study population was 2.80 kg for males and 1.80 kg for females. All the measured parameters had a significant effect on the phenotype, except for body weight, tarsus diameter, and beak width, where the different phenotypes did not show any significant effect; rather all the phenotypes had almost the same values (Table 3). Moreover, Muscovy ducks with spotted white and grey colour were found to have the highest values for the majority of the parameters, while the white-green-black colour exhibited the highest values for the thoracic cage (14.93 cm), thoracic circumference (36 cm), shank circumference (7.30 cm), chest height (12.78 cm), and beak length (5.06 cm). The white-colored ducks displayed the highest value for leg length (9.93 cm).

Again, shank length, tarsus length, tarsus diameter, wing length, and beak width did not show any significant differences between (P < 0.05) sexes.

### 3.2. Qualitative phenotypic traits of Muscovy ducks across agroecological zones in Benin

**Fig 2:**
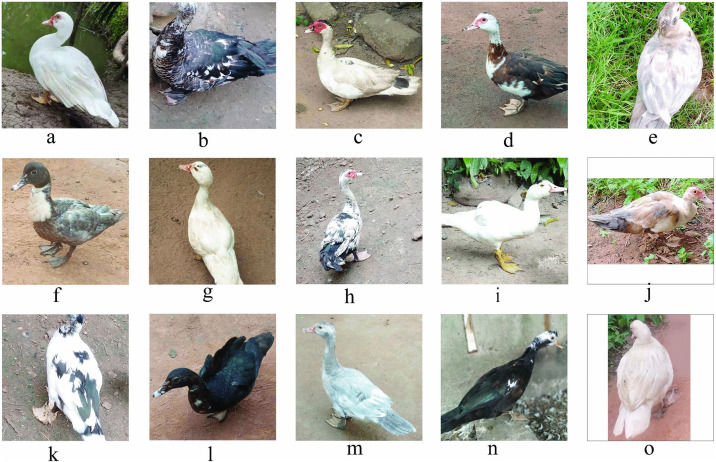
Phenotypic Variation in Plumage Coloration of Muscovy Ducks.

**Fig 3:**
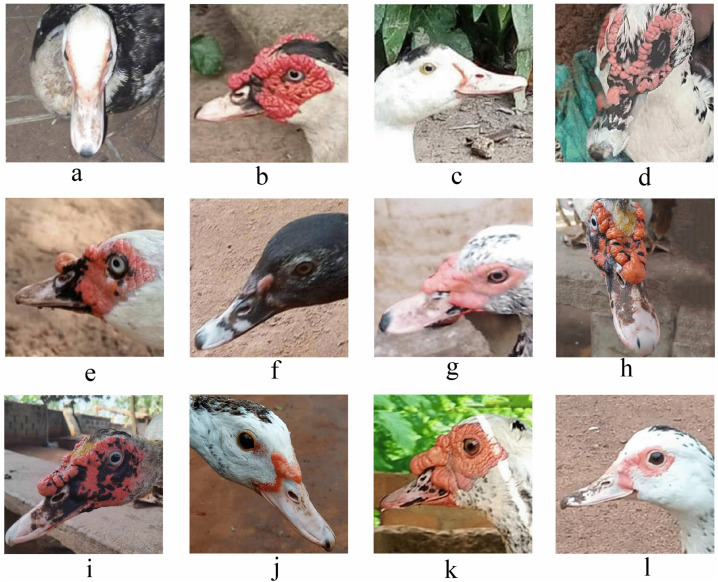
Beak color. The primary distinction observed among the beaks lies in the arrangement of the bean, bill, and nostril, as well as the specific combination of their configurations.

**Fig 4:**
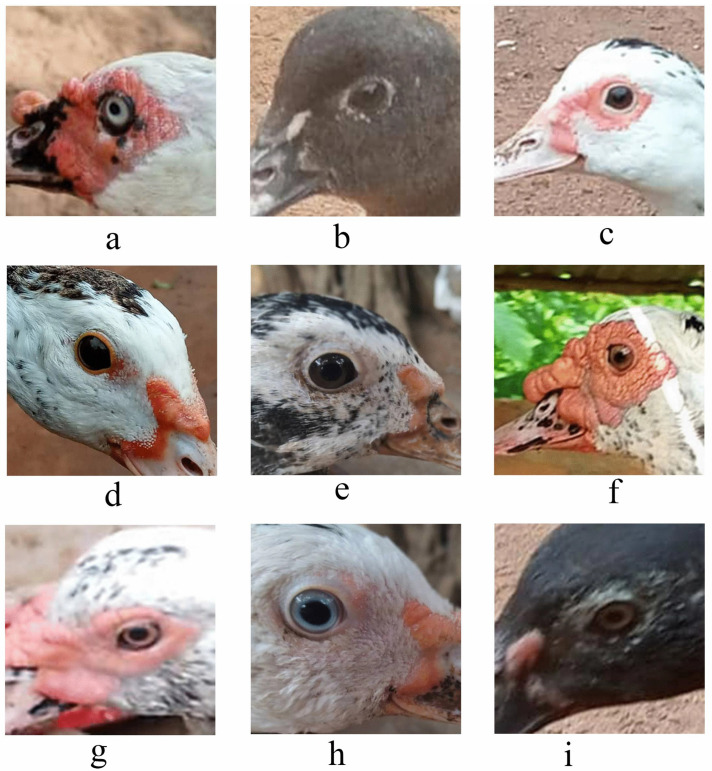
Phenotypic Variation in the Eye Colour of Muscovy Ducks.

**Fig 5:**
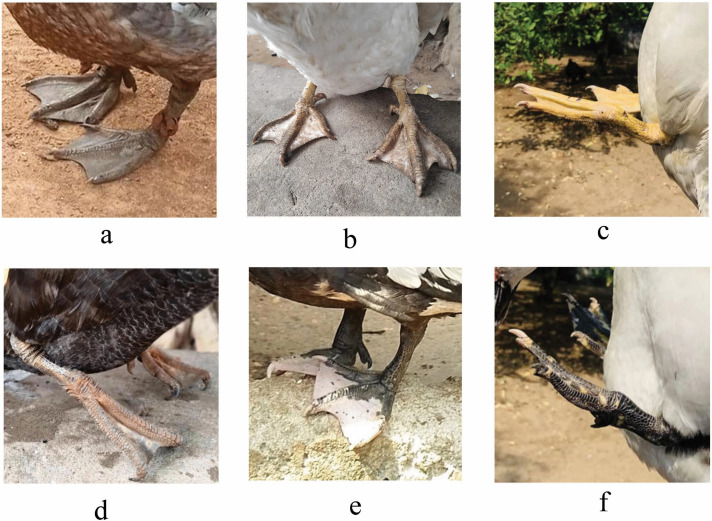
Phenotypic Variation in the Colour Patterns of Muscovy Ducks.

Figs 2–5 and Table 4 summarize the qualitative traits of Muscovy ducks across different agroecological zones. Variations in phenotype were observed in each zone, with distinct modal expressions for various characteristics (P < 0.05). Regarding plum-age patterns, three main categories (pie, solid, and spotted) were identified. Solid plumage patterns accounted for 65% of the total population, with the highest frequency (48.73%) in zone 5. The pie pattern, which was the least common, was absent in Zone 8, while white plumage was the predominant, representing 58% of the population, followed by spotted white and black (23%). Other plumage hues accounted for less than 1%. Zone 5, in particular, had a strong representation of gray-black mottled and spotted white-gray plumages, while zone 6 exhibited exclusive occurrences of green-black-white and white-green-blue combinations. The body carriage of the ducks in the study population was largely horizontal (98%), with only 2% slightly upright (S2 File).

**Table 4:** Qualitative traits of Muscovy duck in the different Zone.

Parameter	Modalities	Overall Count	Zone 5	Zone 6	Zone 8	X²
Plumage Pattern	Pie	6	5 (83.33%)	1 (16.67%)	0 (0.0%)	29.65
	Solid	275	134 (48.73%)	116 (42.18%)	25 (9.09%)	29.65
	Spotted	125	28 (22.4%)	83 (66.4%)	14 (11.2%)	29.65
Plumage Colour	Black	18	9 (50.0%)	4 (22.22%)	5 (27.78%)	59.36
	Gray and black mottled	3	3 (100.0%)	0 (0.0%)	0 (0.0%)	59.36
	Green, black, and white mottled	1	0 (0.0%)	1 (100.0%)	0 (0.0%)	59.36
	Spotted white and black	100	22 (22.0%)	68 (68.0%)	10 (10.0%)	59.36
	Spotted white and gray	2	2 (100.0%)	0 (0.0%)	0 (0.0%)	59.36
	White	246	122 (49.59%)	105 (42.68%)	19 (7.72%)	59.36
	ash	19	4 (21.05%)	10 (52.63%)	5 (26.32%)	59.36
	black piebald	8	5 (62.5%)	3 (37.5%)	0 (0.0%)	59.36
	white green black	8	0 (0.0%)	8 (100.0%)	0 (0.0%)	59.36
	white green blue	1	0 (0.0%)	1 (100.0%)	0 (0.0%)	59.36
Skin Colour	Black	54	10 (18.52%)	43 (79.63%)	1 (1.85%)	36.69
	Blue-Black	23	12 (52.17%)	9 (39.13%)	2 (8.7%)	36.69
	White	312	138 (44.23%)	144 (46.15%)	30 (9.62%)	36.69
	Yellow	17	7 (41.18%)	4 (23.53%)	6 (35.29%)	36.69
Beak Colour	Beige Bill	25	3 (12.0%)	18 (72.0%)	4 (16.0%)	43.26
	Beige Bill with black bean	88	26 (29.55%)	53 (60.23%)	9 (10.23%)	43.26
	Black Bill	19	2 (10.53%)	12 (63.16%)	5 (26.32%)	43.26
	Pink Bill with black Bean	267	135 (51%)	112 (42%)	20 (7%)	43.26
	Pink Bill with white Bean	1	0 (0.0%)	1 (100.0%)	0 (0.0%)	43.26
	Yellow Bill	6	1 (16.67%)	4 (66.67%)	1 (16.67%)	43.26
Eye Colour	Black	159	10 (6.29%)	147 (92.45%)	2 (1.26%)	246.03
	Bleu	1	1 (100.0%)	0 (0.0%)	0 (0.0%)	246.03
	Brown	122	50 (40.98%)	49 (40.16%)	23 (18.85%)	246.03
	White	124	106 (85.48%)	4 (3.23%)	14 (11.29%)	246.03
Shank Colour	Black	91	9 (9.89%)	68 (74.73%)	14 (15.38%)	77.18
	Black speckled with white	3	3 (100.0%)	0 (0.0%)	0 (0.0%)	77.18
	Gray	28	13 (46.43%)	12 (42.86%)	3 (10.71%)	77.18
	Gray and yellow	3	2 (75%)	0 (0.0%)	1 (25%)	77.18
	Pink	1	1 (100.0%)	0 (0.0%)	0 (0.0%)	77.18
	White	40	11 (27.5%)	27 (67.5%)	2 (5.0%)	77.18
	Yellow	238	126 (52.94%)	93 (39.08%)	19 (7.98%)	77.18
	Yellow speckled with black	2	2 (100.0%)	0 (0.0%)	0 (0.0%)	77.18

Note: The zones referenced are as follows: ZAE5 (Central Cotton Zone), ZAE6 (“Terre de Barre” Zone), and ZAE8 (Fisheries Zone).

Eye colour distribution revealed a dominance of black (38.59%), followed by white (30%), brown (29.61%), and blue (less than 1%). White and brown eyes were most frequently observed in zone 8, while black eyes were predominantly found in zone 5. Yel-low shank colour was the most common (58.62%), followed by black (22.41%), white (9.85%), and gray (7%). Minor variations, including black speckled with white, gray-yellow, pink, and yellow speckled with black, were recorded at less than 1% frequencies.

Again, white skin colour was the most prevalent (73%), followed by black (13%) and yellow, which appeared in less than 1% of the population. To evaluate beak colour, we examined the colours of the bill, bean, and nostril and their distribution (Table 2). The most prevalent bill colour was pink (66%), followed by beige (28%), black (19%), and yellow (1%). Pink bill was particularly prominent in zone 5, which contributed over 49.5% of the observations for this trait, while the black bean colour was predominant.

Furthermore, a higher percentage of Muscovy ducks without crests (95%) were found in the study population.

### 3.3. Correlation analysis and spatial representation of morphometric traits in muscovy ducks across different agroecological zones

**Fig 6:**
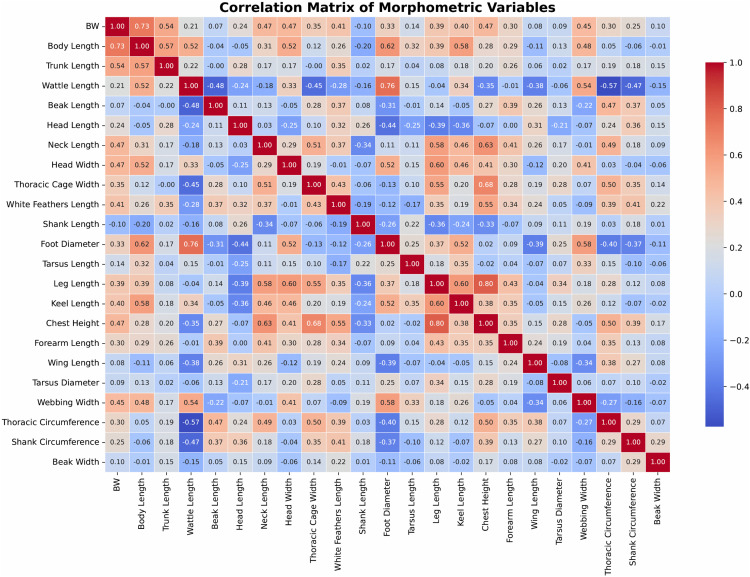
Correlation matrix among morphometric measurement.

The relationship among the features was depicted using the correlation matrix. The analyses revealed strong positive correlations between body weight and body length (0.73), leg length and chest height (r = 0.80), wattle length, and foot diameter (r = 0.76) across all datasets (Fig 6). Specifically, for females, the top correlated features were Leg Length and Chest Height (0.741), Wattle Length and Foot Diameter (0.737), and Head Length and Foot Diameter (−0.687), while for males, the highest correlations were Leg Length and Chest Height (0.883), Wattle Length and Foot Diameter (0.802), and Foot Diameter and Wing Length (−0.793) (S1 Excel).

**Fig 7:**
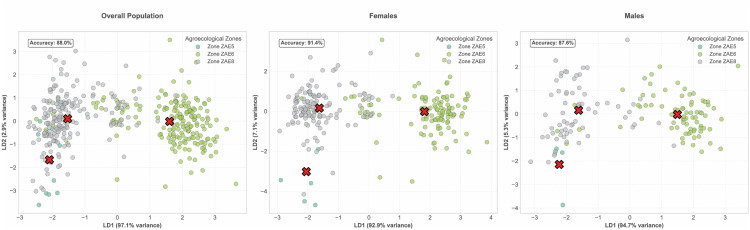
Linear Discriminant Analysis (LDA) of Morphometric Variable in the three Agroecological Zone.

Furthermore, linear discriminant analysis (LDA) was employed to identify the linear combinations of features that best distinguish the zones. As depicted in Fig 7, a clear and distinct pattern emerged from the LDA analysis, enabling the separation of zones based on morphometric traits for both female and male and overall. By evaluating the coefficients derived from LDA, the importance of each feature in zone dis-crimination was assessed and further analyzed through spatial projections.

As displayed in Table 5, for females, the most discriminating features are Leg Length (7.73), Chest Height (4.40), and Body Length (3.61), for males, the top features include Wattle Length (7.69), Chest Height (3.29), and Keel Length (3.21) while Chest Height was identified as a significant discriminative parameter among the sexes, indicating its crucial role in differentiating the zones.

**Table 5:** Discriminatory feature coefficients from linear discriminant analysis for female and male groups.

Feature	Female Coefficient	Male Coefficient
Leg Length	7.73	2.31
Chest Height	4.4	3.29
Body Length	3.61	0.28
Beak Length	3.37	1.57
Forearm Length	2.9	1.72
Tarsus Length	2.41	0.72
Tarsus Diameter	2.26	0.99
White Feathers Length	2.2	1.31
Thoracic Cage Width	2.16	0.99
Head Length	2.13	0.38
Webbing Width	2.03	0.65
Foot Diameter	1.86	0.37
Thoracic Circumference	1.85	1.56
Trunk Length	1.74	1.11
Shank Length	1.64	1.32
Keel Length	1.55	3.21
Wattle Length	1.49	7.69
Head Width	1.34	3.06
BW	1.12	1.93
Neck Length	1.04	2.28
Shank Circumference	1.04	1.96
Wing Length	0.67	1.96
Beak Width	0.62	2.55

The cross-validation results indicate that the model achieves an average accuracy of 80.78% for females, with relatively high variability (±28.85%). The model performs slightly better for males, with an accuracy of 83.45% and lower variability (±12.79%). The confusion matrix and classification report (Supplementation file S1–S4 Table) confirm these findings. For females, the model achieves an overall accuracy of 91%, with high precision and recall for ZAE 8 (f1-score: 0.92) and strong performance for ZAE 6 (f1-score: 0.92), though ZAE5 shows moderate performance (f1-score: 0.62). Similarly, for males, the model achieves an accuracy of 88%, with a strong performance in ZAE 6 (f1-score: 0.92) and ZAE 8 (f1-score: 0.86), while ZAE 5 demonstrates weaker results (f1-score: 0.29), likely due to its smaller sample size. These results highlight the model’s ability to reliably distinguish between zones, particularly for the dominant ZAE, while indicating some limitations in predicting minority ZAE. The above findings were furthermore supported by the PCA results (S5 Table). The first two principal components explained 22.90% and 20.15% of the variance in females and 36.51% and 25.42% in males, respectively, indicating greater morphological variability among males (S1 File).

## 4. Discussion

Morphometric characterization of ducks, particularly in regions where such studies are scarce, is crucial for understanding breed populations and establishing strategies for conservation and the sustainable use of genetic resources [17].

Biometry is a useful tool for measuring phenotypic variation within local populations, which is critical for optimizing genetic resources and encouraging sustainable breeding strategies [11].

In this study, the greater predominance of females can be attributed to the fact that breeders use female ducks to incubate eggs of other species, as ducks are known to be efficient natural incubators in breeding.

This was confirmed in our previous study [8] and in the study by [18] on the indigenous Muscovy ducks breed in the backyard (Brazzaville, the Congo Republic). In contrast, [17] reported a higher proportion of males, which was attributed to the practices of small-scale duck farmers, who prefer male ducks due to their faster growth rates and greater profitability for income generation. In addition, the research revealed a high percentage of crested (95%) versus crested (5%) Muscovy ducks in the study population. In line with these trends, previous studies have also found that the absence of crests (69.65%) was higher than the presence of crests (30.35%) in the south-western region of Nigeria [17]; however, [19] found that Muscovy ducks in south-eastern Nigeria were crestless. The predominant yellow shank colour (58.62%) found in this study is corroborated by [20] and [21] in Cambodia.

Blue eye colour was observed for the first among the duck population alongside black (38.59%), white (30%) and brown (29.51%). In contrast, previous studies have reported brown to be the dominant eye colour among Nigerian Muscovy ducks [17,19]. This rare blue eye colour may have resulted from a genetic mutation or recessive trait, demonstrating the genetic diversity within domestic duck populations [22]. Although black, white, and brown eye colours align with broader phenotypic trends, grey eye colour was absent in our study population. These results highlight regional variation in phenotypic traits and emphasize the influences of genetics, environment, and possibly random breeding in determining eye coloration.

Moreover, the most common skin colour found in this study was white (73%), followed by black (13%), while yellow (less than 1%) was extremely uncommon. In a similar study, [20] found that Muscovy ducks typically had white and yellow skin colours, whereas Bangladesh duck species had black or black with a yellow tint [23]. Muscovy ducks’ skin colour differences may be caused by genetics, similar to those in other waterfowl, where certain genetic pathways regulate pigmentation [24,25]. A deeper understanding of the genetic processes behind these colour variations may be possible through future molecular research, which could be helpful for selective breeding initiatives.

A predominance of pink bill colour (66%), followed by beige (28%) and black (19%), with yellow being the least common (1%), was found in this study. This aligns with the observations of [17] and [19], who reported pink-white as the dominant bill colour among Muscovy ducks in southwestern and southeastern Nigeria, respectively. Conversely, [26] and [20] noted black-yellow and black as dominant bill colours in ducks from other regions of Nigeria, demonstrating geographical variation in phenotypic traits. The predominance of black bean colour in this study corroborates findings by [21] in Nigeria, while [19] identified white as the dominant bean colour in some regions, highlighting the role of sampling coverage in trait variability. Evolutionary biology suggests that colour polymorphism within species can contribute to phenotypic diversity, as seen here, with such traits potentially evolving as adaptive mechanisms in local Muscovy ducks [27]. Furthermore, adaptive traits like colour diversity may reflect evolutionary responses to environmental pressures, including the need for tropical livestock to withstand climatic challenges while maintaining productivity [28]. These findings underscore the influence of both genetic and ecological factors on phenotypic diversity.

The study equally revealed white as the predominant plumage colour (58%) and this agrees with [13], who reported similar results among domestic geese raised in northern Benin. Nevertheless, studies conducted in Congo [18], North central Nigeria [29], and Vietnam [30] all reported the black feathered as the most popular duck strain in the native population.

The dominance of the white feather colour found in this study could be linked to the morals and customs of local populations who exploit many of these phenotypes for cultic purposes and, therefore, exert positive selection. White is a symbol of purity in both Africa and Europe, and breeders’ preference for white makes white feathers more popular [8]. Furthermore, the interaction of evolutionary forces such as selection, migration, mutation, and local environmental pressures likely contributes to the variation in plumage coloration, as evidenced by the presence of various other genetic colour variants in Muscovy ducks.

Body carriage for Muscovy local ducks was predominantly horizontal, with a few showing slight upright postures (S2 File). The findings of this study follow the FAO [9] guidelines, which describe the body carriage of Aylesbury ducks and Muscovy ducks as horizontal, while the body carriage of Indian Runner and Bali ducks is upright. The slightly upright profile found in ducks could possibly be the result of interbreeding between breeds caused by unintentional human intervention. For instance, when duck farmers migrate to new locations, it takes long for their flocks to resettle. As a result of this migration, existing settlers and new ones could exchange or mate birds. Another explanation for the intermixing is that household duck farmers purchase ducks to repopulate their flock from another location or neighboring duck markets.

### 4.1. Biometric characteristics of Muscovy duck in Benin

Sexual dimorphism in morphometric traits was evident in this study, with males showing consistently higher values across most measured parameters (P < 0.05), particularly in body weight, body length, thoracic dimensions, and head size. Among the Agroecological zones, ZAE 6 exhibited the highest biometric values overall.

Consistent with [17], who reported Nigerian Muscovy drakes weighing between 2.17–2.87 kg and hens between 1.33–1.90 kg, Benin Muscovy ducks in this study averaged 2.80 kg for males and 1.80 kg for females. This aligns with [31], while studies from southeastern Nigeria [19] and Vietnam [30] reported even higher weights (up to 3.36 kg in males), suggesting geographical variation influenced by environmental and genetic factors.

Body length measurements align with findings from North-West Ethiopia and North Central Nigeria [29,32], where male lengths ranged from 56.88 cm to 47.52 cm, com-pared to 54.59 cm to 49.02 cm in females. In contrast, smaller lengths were observed in Nigeria’s ecological zones [33], while [34,35] in India and [19] in Nigeria reported slightly larger values overall. Plumage colour also emerged as a factor, as noted by [36] in Papua New Guinea, further supporting the interplay between genetic traits and environmental adaptation in shaping body conformation [25].

Thoracic traits such as cage width, chest height, and circumference were generally larger in males (13.50 cm, 10.81 cm, and 28.07 cm, respectively), a pattern corroborated by [15,19,32]. These traits are likely influenced by physiological differences driven by sexual selection, as also noted in prior literature [19].

Beak measurements showed only minor sex-based differences, with males slightly exceeding females in width (2.48 cm vs. 2.40 cm) and length (4.15 cm vs. 4.12 cm). This subtle variation, supported by [33–35], contrasts with the more pronounced dimorphism seen in thoracic and overall body size.

Neck length differences followed a similar pattern, with males averaging 13.61 cm compared to 11.23 cm in females. Studies from Bangladesh [23], Indonesia [37], and Nigeria [33] echo this trend, highlighting how Agro-ecological conditions and plumage colour variations, as observed in [36], contribute to inter-population variability.

Shank circumference and hock length also demonstrated dimorphism, with male hocks averaging 10.43 cm and females 9.40 cm. This aligns with observations in [19,38], reflecting underlying biological conformation [25]. Head length, another sexually di-morphic trait, was larger in males (7.71 cm vs. 6.29 cm), consistent with findings in [15,35], and may relate to functional roles in feeding efficiency and dominance.

Lastly, while leg length differences were evident (males: 10.43 cm; females: 9.40 cm), the disparity was smaller than for traits like body length or thoracic circumference. This suggests leg length is less subject to sexual selection pressures, as noted by [13,33], and may play a more neutral role in dimorphism compared to traits more closely tied to reproductive fitness.

### 4.2. Correlation among the features and spatial disposition

The correlation analysis revealed strong positive associations between body weight and body length, leg length and chest height, and wattle length and foot diameter across all datasets, with females showing moderate to strong correlations and males exhibiting slightly stronger associations. These results are consistent with those of [32] and [19], who found similar significant correlations between body weight and linear body measurements like chest circumference. The observed high correlation between body weight and body length (0.74) in females aligns with [17], who reported a similar trend in ducks.

The LDA analysis, used to determine the most discriminating parameters in separating different agroecological zones, revealed that in females, leg length, chest height, and body length were the most discriminating characteristics. In males, on the other hand, wattle length, chest height, and keel length were identified as the main discriminating characteristics.

In this study, chest height was identified as the most discriminating parameter, regardless of sex. Indeed, previous studies have already identified chest height as a reliable predictor of live body weight in poultry [39], cattle [40], and small ruminants [41], confirming that this parameter can be considered a key morphological factor for assessing body mass and structural development. This makes it an essential trait for distinguishing variations between different agroecological zones and between the sexes.

This study can be applied in the field, where simple, non-invasive measurements can be used for effective weight prediction and informed breeding decisions, thus sup-porting sustainable livestock management strategies.

## 5. Conclusion

This study characterized the morphological and phenotypic diversity of Muscovy ducks across three agro-ecological zones in southern Benin, highlighting significant variations influenced by genetic and environmental factors. Sexual dimorphism was evident, with males exhibiting larger body dimensions, while the predominance of females reflected their role in incubation practices. Key traits such as plumage, eye, and bill colour varied regionally, indicating genetic diversity and potential adaptive responses to local conditions. The strong correlations between body weight and morphometric traits suggest that simple, non-invasive measurements can be used for weight estimation and breeding selection. These findings provide a foundation for future genetic improvement and conservation strategies to optimize local duck production.

## Supporting information

S1 Table Female Classification Report. (DOCX)

S2 Table Confusion Matrix for Female. (DOCX)

S3 Table Male Classification Report. (DOCX)

S4 Table Confusion Matrix for Female. (DOCX)

S1 File PCA of Morphometric Variable. (DOCX)

S2 File Body carriage in ducks. (DOCX)

S5 Table PCA Loadings and Variance Analysis by Sex. (DOCX)

S1 Excel Correlation matrix among Male, Female for the morphometric Parameters. (XLSX)
