# Supplementary material for: Phenotypic and morphometric characterization of local muscovy ducks raised in West Africa, Benin
Source: PLoS One. 2025 Dec 31;20(12):e0338829. doi: 10.1371/journal.pone.0338829 (PMC12755831; doi:10.1371/journal.pone.0338829)
Supplement: S1 Table — (DOCX) [file pone.0338829.s001.docx]

Table S1: Female Classification Report:

|  | precision | recall | f1-score | support |
| --- | --- | --- | --- | --- |
| ZAE5 | 0.57 | 0.67 | 0.62 | 6 |
| ZAE 6 | 0.99 | 0.86 | 0.92 | 121 |
| ZAE 8 | 0.87 | 0.98 | 0.92 | 128 |
|  |  |  |  |  |
| accuracy |  | | 0.91 | 255 |
| macro avg | 0.81 | 0.83 | 0.82 | 255 |
| weighted avg | 0.92 | 0.91 | 0.91 | 255 |
