# Supplementary material for: Phenotypic and morphometric characterization of local muscovy ducks raised in West Africa, Benin
Source: PLoS One. 2025 Dec 31;20(12):e0338829. doi: 10.1371/journal.pone.0338829 (PMC12755831; doi:10.1371/journal.pone.0338829)
Supplement: S2 Table — (DOCX) [file pone.0338829.s002.docx]

Table S2: Confusion Matrix for Female

| Predicted\True | ZAE = 5 | ZAE = 6 | ZAE = 8 |
| --- | --- | --- | --- |
| ZAE = 5 | 4 (TP) | 0 (FP) | 2 (FN) |
| ZAE = 6 | 1 (FN) | 104 (TP) | 16 (FP) |
| ZAE = 8 | 2 (FN) | 1 (FP) | 125 (TP) |
