# Supplementary material for: Phenotypic and morphometric characterization of local muscovy ducks raised in West Africa, Benin
Source: PLoS One. 2025 Dec 31;20(12):e0338829. doi: 10.1371/journal.pone.0338829 (PMC12755831; doi:10.1371/journal.pone.0338829)
Supplement: S3 Table — (DOCX) [file pone.0338829.s003.docx]

Table S3: Male Classification Report

|  | precision | recall | f1-score | Support |
| --- | --- | --- | --- | --- |
| ZAE5 | 0.33 | 0.25 | 0.29 | 4 |
| ZAE 6 | 0.91 | 0.92 | 0.92 | 76 |
| ZAE 8 | 0.86 | 0.86 | 0.86 | 65 |
|  |  |  | 0.88 | 145 |
| accuracy |  |  | 0.88 | 145 |
| macro avg | 0.70 | 0.68 | 0.69 | 145 |
| weighted avg | 0.87 | 0.88 | 0.87 | 145 |
