# Supplementary figures and images for: Phenotypic and morphometric characterization of local muscovy ducks raised in West Africa, Benin
Source: PLoS One. 2025 Dec 31;20(12):e0338829. doi: 10.1371/journal.pone.0338829 (PMC12755831; doi:10.1371/journal.pone.0338829)

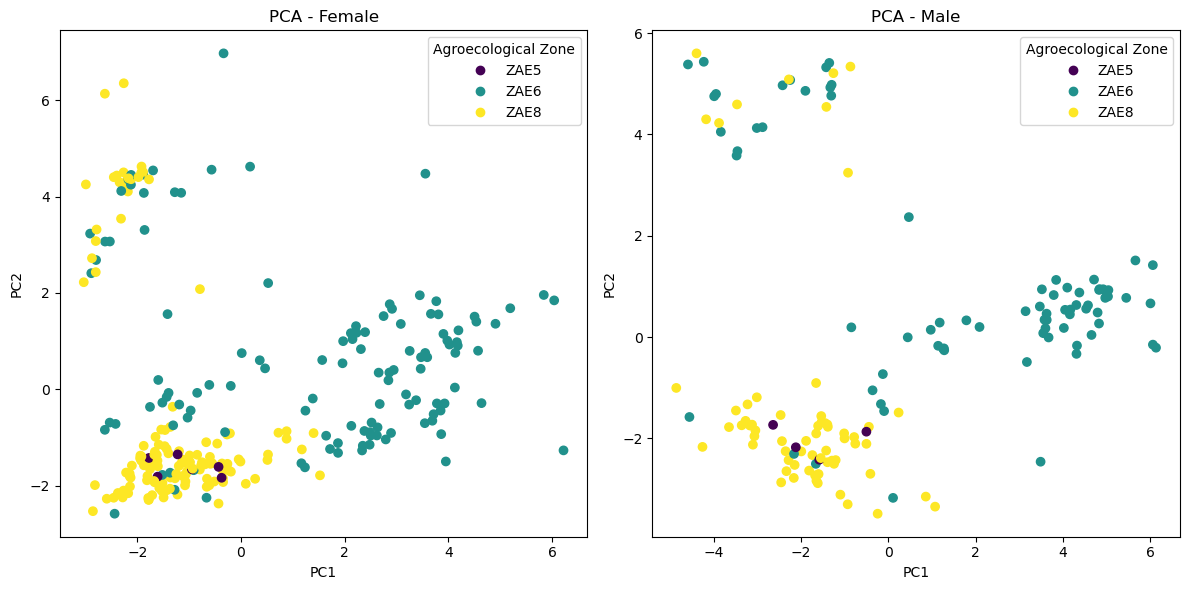


**SFF1:** PCA of Morphometric Variable

Supplement: S1 File — (DOCX) [file pone.0338829.s005.docx]

| **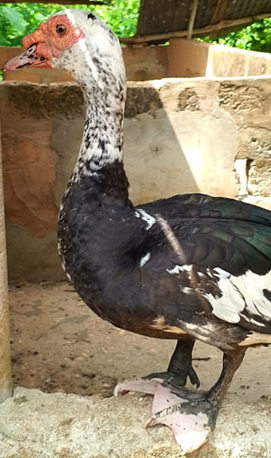** | **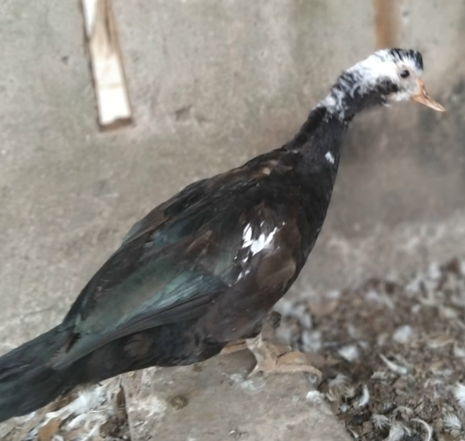** |
| --- | --- |
| **Horizontal Body Profile** | **Slightly upright Body Profile** |

**SFF2**: Body carriage in ducks

Supplement: S2 File — (DOCX) [file pone.0338829.s006.docx]
