# Supplementary material for: Phenotypic and morphometric characterization of local muscovy ducks raised in West Africa, Benin
Source: PLoS One. 2025 Dec 31;20(12):e0338829. doi: 10.1371/journal.pone.0338829 (PMC12755831; doi:10.1371/journal.pone.0338829)
Supplement: S5 Table — (DOCX) [file pone.0338829.s007.docx]

**Table S5 : PCA Loadings and Variance Analysis by Sex**

| Variable | All | | | Male | | | Female | | |
| --- | --- | --- | --- | --- | --- | --- | --- | --- | --- |
|  | **PC1** | **PC2** | **PC3** | **PC1** | **PC2** | **PC3** | **PC1** | **PC2** | **PC3** |
| BW | 0.31 | -0.06 | -0.33 | 0.31 | -0.05 | -0.18 | 0.28 | 0.08 | -0.37 |
| Body Length | 0.28 | -0.23 | -0.25 | 0.27 | -0.23 | 0.03 | 0.29 | -0.08 | -0.38 |
| Trunk Length | 0.18 | -0.03 | -0.45 | 0.16 | -0.2 | -0.12 | 0.06 | 0.15 | -0.41 |
| Wattle Length | 0 | -0.41 | -0.17 | 0.05 | -0.36 | -0.06 | 0.07 | -0.39 | -0.19 |
| Beak Length | 0.11 | 0.25 | 0.04 | 0.01 | 0.31 | 0.13 | 0.1 | 0.23 | -0.15 |
| Head Length | -0.03 | 0.22 | -0.47 | -0.13 | 0.15 | -0.49 | -0.22 | 0.24 | -0.27 |
| Neck Length | 0.31 | 0.08 | 0.1 | 0.27 | 0.13 | 0.13 | 0.23 | 0.17 | 0.19 |
| Head Width | 0.25 | -0.2 | 0.05 | 0.32 | -0.12 | -0.14 | 0.25 | -0.13 | 0.07 |
| Thoracic Cage Width | 0.26 | 0.18 | 0.13 | 0.2 | 0.24 | -0.2 | 0.21 | 0.22 | 0.26 |
| White Feathers Length | 0.23 | 0.2 | -0.15 | 0.23 | 0.21 | -0.11 | 0.06 | 0.27 | -0.19 |
| Shank Length | -0.11 | 0.06 | -0.24 | -0.26 | 0.03 | -0.28 | 0 | 0.05 | -0.24 |
| Foot Diameter | 0.15 | -0.39 | 0.02 | 0.15 | -0.32 | 0.01 | 0.27 | -0.31 | 0.01 |
| Tarsus Length | 0.09 | -0.14 | 0.02 | -0.03 | -0.07 | 0.33 | 0.28 | -0.09 | -0.12 |
| Leg Length | 0.34 | -0.04 | 0.29 | 0.34 | 0.06 | 0.12 | 0.36 | 0.05 | 0.26 |
| Keel Length | 0.28 | -0.18 | 0.11 | 0.22 | -0.1 | 0.29 | 0.36 | -0.06 | 0.02 |
| Chest Height | 0.34 | 0.14 | 0.17 | 0.31 | 0.18 | 0.03 | 0.26 | 0.24 | 0.25 |
| Forearm Length | 0.24 | 0.06 | -0.01 | 0.2 | 0.1 | -0.09 | 0.23 | 0.13 | -0.12 |
| Wing Length | 0.05 | 0.25 | -0.09 | -0.04 | 0.34 | -0.09 | -0.01 | 0.22 | -0.08 |
| Tarsus Diameter | 0.13 | -0.02 | 0.19 | 0.24 | 0 | -0.16 | 0.17 | 0.04 | 0.12 |
| Webbing Width | 0.11 | -0.28 | -0.21 | 0.08 | -0.22 | -0.45 | 0.19 | -0.27 | -0.15 |
| Thoracic Circumference | 0.2 | 0.28 | 0.01 | 0.06 | 0.29 | 0.22 | 0.13 | 0.34 | 0.04 |
| Shank Circumference | 0.12 | 0.27 | -0.16 | 0.09 | 0.32 | -0.14 | 0.01 | 0.29 | -0.11 |
| Beak Width | 0.06 | 0.11 | -0.13 | 0.24 | 0.06 | -0.07 | -0.02 | 0.15 | -0.06 |
| % Variance Explained | 24.71 | 20.35 | 9.48 | 31.06 | 28.43 | 8.04 | 22.9 | 20.15 | 10.02 |

Results demonstrate that Leg Length, Chest Height, Keel Length, Foot Diameter and Wattle Length are main contributors to morphometric variation, which differs across sexes and agroecological zones (Table 4). Specifically, in females, Leg Length (0.36) and Keel Length (0.36) contributed most to PC1, while Wattle Length (-0.39) and Thoracic Circumference (0.34) contributed most to PC2. In opposite, for males, Leg Length (0.34) and Head Width (0.32) contributed to PC1, whereas Wattle Length (-0.36) and Wing Length (0.34) contributed to PC2, reflecting head and flight-related traits. Among all individuals combined, PC1 was dominated by Leg Length and Chest Height (0.34), PC2 by Wattle Length and Foot Diameter (-0.41), which indicates that structural size and body proportions contribute consistently.
